# Prognostic factors and treatment outcomes of spinal osteosarcoma: Surveillance, epidemiology, and end results database analysis

**DOI:** 10.3389/fonc.2023.1083776

**Published:** 2023-03-01

**Authors:** Jing Wang, Xiang-zhi Ni, Ming-lei Yang, Xing Huang, Shu-ming Hou, Cheng Peng, Jia-shi Cao, Tie-Long Liu

**Affiliations:** Department of Orthopaedic Oncology, Spinal Tumor Center, Shanghai Changzheng Hospital, Naval Medical University, Shanghai, China

**Keywords:** spinal osteosarcoma, epidemiology, SEER, survival, prognosis

## Abstract

**Objective:**

Spinal osteosarcoma is a rare osseous neoplasm. The aim of this study is to make a comprehensive analysis of the demographic features, clinicopathologic characteristics and factors affecting prognosis of spinal osteosarcoma using the Surveillance, Epidemiology and End Results (SEER) database.

**Methods:**

SEER data were reviewed to identify patients diagnosed with spinal osteosarcoma between 1975 and 2016 and determine their overall survival (OS) and disease-specifc survival (DSS). Univariate and multivariate analyses were performed using the Cox-regression proportional hazards model and Kaplan-Meier method.

**Results:**

A total of 668 patients (53.1% males) with spinal osteosarcoma were identified. The mean age at diagnosis was 45.2 years, including 67.5% patients younger than 60 years. The median OS of these patients was 15 months, and the 5-year OS was 16.8%. Multivariate analysis showed that age ≥60 year (HR=2.271, p = 0.008), high grade (HR=1.323, p = 0.008), regional stage (HR=1.658, p = 0.017), metastasis stage (HR=3.045, p < 0.001) and no-surgery treatment (HR=1.761, p < 0.001) were adversely associated with OS; gender (HR=0.657, p = 0.044), tumor grade (HR=1.616, p = 0.006), tumor stage (HR=3.329, p = 0.011; HR=7.983, p < 0.001) and radiotherapy (HR=0.606, p = 0.031) were independent prognostic factors affecting DSS.

**Conclusion:**

Based on SEER data analysis, male, high tumor grade, regional stage, metastasis stage and radiotherapy are independent predictors of poor survival of patients with spinal osteosarcoma. The clinical treatment of spinal osteosarcoma still faces serious challenges. Future research should focus on the clinical impact and survival outcomes of the emerging targeted and immune therapies for the sake of improving the survival stalemate of spinal osteosarcoma.

## 1 Introduction

Osteosarcoma is the most common malignant bone sarcoma, accounting for 0.5% of all malignant tumors (1, 2). Osteosarcoma derives from mesenchymal stem cells, characterized by the proliferation of neoplastic cells and the formation of immature bone or osteoid tissues (3). While, spinal osteosarcoma is relatively rare, accounting for 3-5% of all spinal malignancies (4, 5). It mainly affects the sacral region, followed by the lumbar and thoracic segments (6). Given the complex anatomic sites and the severe neurological lesions, clinical treatment of spinal osteosarcoma has always been a challenge, with relatively a worse prognosis as compared with limb osteosarcoma (7, 8).

The current standard treatment for osteosarcoma includes surgical resection of the primary tumor after neoadjuvant chemotherapy, surgical resection of all clinically significant metastases, and postoperative adjuvant chemotherapy. By this way, the 5-year overall survival (OS) rate of patients with osteosarcoma has increased gradually from 60% to 80% (9, 10). Despite the great success achieved in the treatment of osteosarcoma, the treatment of spinal osteosarcoma still faces considerable challenges due to high recurrence, metastasis susceptibility and a mortality rate (11, 12). Due to its rarity, most studies on spinal osteosarcoma are limited to small case reports from individual institutions (13). More research based on larger populations is required to estimate the survival of patients with spinal osteosarcoma and identify factors affecting their survival prognosis.

In 1973, the surveillance, epidemiology and end results (SEER) registration maintained by the National Cancer Institute (NCI) began collecting cancer-related information, and since then it has covered 30% of the total US population and become a comprehensive source of population-level cancer data (14). The sample size of the SEER is larger than that of any single institution and most multi-institutions. To the best of our knowledge, so far there is no multivariate regression analysis on the treatment, confounding factors and independent prognostic factors affecting the prognosis of spinal osteosarcoma. The aim of the present study is to make a comprehensive analysis of the demographic features, clinicopathologic characteristics and factors affecting prognosis of spinal osteosarcoma using the Surveillance, Epidemiology and End Results (SEER) database, and propose treatment strategies for the treatment of this malignancy.

## 2 Materials and methods

### 2.1 Ethics approval

A population-based search was made of patients diagnosed with spinal osteosarcoma by using the case-listing session protocol from the NCI’s SEER 18 database [www.seer.cancer.gov]. The database is publicly available online, and shows no personal identification, so it does not need the approval from the internal review board approval.

### 2.2 Research population

Data of the patients diagnosed with spinal osteosarcoma between 1975 and 2016 were reviewed, and this is the widest date range available in the latest version of the software. Firstly, the primary tumors on the spine, C41.2 (vertebral column) and C41.4 (pelvic bones, sacrum, coccyx, and associated joints), were identified by site-specific codes. Secondly, the Histologic International Classification of Diseases for Oncology, 3rd edition (ICD-O-3) was used to identify the patients with osteosarcoma (ICD-O-3 histologic type: 9180-9187, 9192-9195), including osteosarcoma NOS, chondroblastic osteosarcoma, fibroblastic osteosarcoma, telangiectatic osteosarcoma, osteosarcoma in Paget’s disease, small cell osteosarcoma, central osteosarcoma, intraosseous well-differentiated osteosarcoma, periosteal osteosarcoma, high grade surface osteosarcoma, and parosteal osteosarcoma. Exclusion criteria were patients who were diagnosed solely based on the clinical findings or imaging, confirmed by the death certificate or autopsy, received unknown treatment method, and/or lacked survival information or survival time missing less than 1 month. Finally, 668 patients diagnosed with spinal osteosarcoma were selected and included in this work.

### 2.3 Analytic variables

The assessed predictor variables from the SEER database included demographics, disease stage, histologic subtype, tumor grade, tumor size, surgical treatment, radiotherapy, chemotherapy, survival months, and cause of death of the patient. The primary outcome was defined as the time (in months) of OS from diagnosis to death with any cause and the time from diagnosis to death with disease-specific survival (DSS) specific to a cancer-related diagnosis.

### 2.4 Statistical analysis

Epidemiological description and survival statistics were given to all variables. All statistical analyses were performed by SPSS software (ver. 21.0; SPSS Inc., Chicago, IL, USA). Median survival and OS were calculated by Kaplan-Meier method. Variables with *p* < 0.2 in univariate analysis were subjected to multivariate analysis. The independent predictors of OS and DSS obtained from multivariate analysis were verified by Cox proportional hazards regression model. The hazard ratio (HR) and the corresponding 95% confidence interval (CI) were used to indicate the impact of each factor on OS and DSS. *p* < 0.05 was considered statistical significance.

## 3 Results

Finally, 668 patients diagnosed with spinal osteosarcoma were selected and included in this work from the database from 1975 to 2016 (**Table 1**), in whom 451 patients (67.5%) were younger than 60 years. Demographically, 53.1% patients were males, and 81.1% patients were White. In addition, 67.7% of patients were diagnosed after 2000. Histologically, the most common tumor grade of differentiation was grade III–IV (51.4%) and the most common histological subtype was osteosarcoma NOS (68.7%), followed by chondroblastic osteosarcoma (18.7%), fibroblastic osteosarcoma (4.5%), osteosarcoma with Paget disease (4.3%), telangiectatic osteosarcoma (1.6%), small cell osteosarcoma (1%), and other rare histological subtypes. The tumor stage was known in 68.9% cases, and most cases presented with regional invasive diseases (29.5%). Tumor size information was available in 48.6% cases, with three groups divided. As for the treatment methods for spinal osteosarcoma, local surgery was applied in more than one-third (39.4%) of the patients, chemotherapy in about two-thirds (67.4%) of the patients, and radiotherapy in 208 patients (31.1%). Ultimately, 339 patients (50.7%) died of osteosarcoma.

**Table 1:** Demographic and clinical characteristics of spinal osteosarcoma patients identified in the SEER database from 1975 to 2016.

Characteristic	Definition	Number of cases	Percentage (%)
***Age (years)***	<60	458	68.6
	≥60	210	31.4
***Gender***	Female	313	46.9
	Male	355	53.1
***Race***	White	542	81.1
	Black	80	12.0
	Other	46	6.9
***Decade of diagnosis***	1970s	37	5.5
	1980s	72	10.8
	1990s	107	16.0
	≥2000s	452	67.7
***Grade***	I-II	44	6.5
	III-IV	343	51.4
	Unknow	281	42.1
***Histological type***	Osteosarcoma, NOS	457	68.7
	Chondroblastic osteosarcoma	125	18.7
	Fibroblastic osteosarcoma	30	4.5
	Osteosarcoma with Paget disease	29	4.3
	Telangiectatic osteosarcoma	11	1.6
	Small cell osteosarcoma	7	1.0
	Central osteosarcoma	5	0.7
	Intraosseous well differentiated osteosarcoma	2	0.3
	Periosteal osteosarcoma	1	0.1
	High grade surface osteosarcoma	1	0.1
***Tumor Stage***	localize	104	15.6
	regional	197	29.5
	metastasis	159	23.8
	unknow	208	31.1
***Tumor size (mm)***	≤50	51	7.6
	50~100	109	16.3
	≥100	165	24.7
	unknow	343	51.4
***Surgery***	Yes	263	39.4
	No	209	31.3
	unknow	196	29.3
***Chemotherapy***	Yes	450	67.4
	No/unknow	218	32.6
***Radiotherapy***	Yes	208	31.1
	No/unknow	460	68.9
***Dead from cancer***	Yes	339	50.7
	No	329	49.3
***Dead***	Yes	522	78.1
	No	146	21.9

The results of univariate analysis in variables associated with survival of the patients with spinal osteosarcoma are shown in **Tables 2**, **3**, indicating that patients over 60 years of age had significantly worse prognosis than those under 60 years of age (HR = 2.550, *p* < 0.001; HR = 1.719, *p* < 0.001). Gender and race had no significant correlation with OS or DSS. A more recent decade of diagnosis was associated with the improved DSS (HR = 0.985, *p* < 0.001) for spinal osteosarcoma. Both OS and DSS varied with different tumor grades, and a higher tumor grade predicted a worse prognosis (HR = 1.458, *p* < 0.001; HR = 1.447, *p* < 0.001). OS and DSS also varied with different tumor stages, and metastasis predicted a worst prognosis. Similarly, a larger tumor size was associated with worse OS and DSS (HR = 1.215, *p* = 0.033; HR = 1.325, *p* = 0.012). OS and DSS in patients who received surgical treatment were significantly better than those in non-surgical patients (HR = 2.651, *p* < 0.001; HR = 2.285, *p* < 0.001). Chemotherapy was significantly associated with OS (HR = 0.827, *p* = 0.043) but not with DSS. However, patients who received radiotherapy had worse OS and DSS than those who did not (HR = 0.615, *p* < 0.001; HR = 0.542, *p* < 0.001).

**Table 2:** Univariate and multivariate analysis for clinical factors associated with OS of spinal osteosarcoma patients.

Characteristic	Univariate analysis			Multivariate analysis		
	HR	95% CI	P value	HR	95% CI	P value
*Age(years)*						
<60	1			1		
≥60	2.550	2.122~3.065	<0.001	2.271	1.650~3.128	0.008
*Gender*						
Male	1					
Female	0.913	0.769~1.085	0.303			
*Race*						
White	1					
Black	1.046	0.800~1.369	0.740			
Other	1.128	0.809~1.572	0.477			
***Decade of diagnosis***	0.945	0.877~1.019	0.140			
***Grade***	1.458	1.237~1.718	<0.001	1.323	1.075~1.628	0.008
*Histological type*						
Osteosarcoma, NOS	1					
Chondroblastic osteosarcoma	0.796	0.632~1.003	0.053			
Other	0.735	0.563~0.960	0.024			
*Tumor Stage*						
localize	1			1		
regional	2.408	1.562~3.711	< 0.001	1.658	1.096~2.507	0.017
metastasis	5.221	3.353~8.129	< 0.001	3.045	1.917~4.836	< 0.001
***Tumor size***	1.215	1.016~1.453	0.033			
*Surgery*						
Yes	1			1		
No	2.651	2.130~3.299	< 0.001	1.761	1.272~2.438	< 0.001
*Chemotherapy*						
Yes	1					
No/unknow	1.209	1.006~1.454	0.043			
*Radiotherapy*						
Yes	1					
No/unknow	0.619	0.516~0.743	< 0.001			

**Table 3:** Univariate and multivariate analysis for clinical factors associated with DSS of spinal osteosarcoma patients.

Characteristic	Univariate analysis			Multivariate analysis		
	HR	95% CI	P value	HR	95% CI	P value
*Age(years)*						
<60	1					
≥60	1.719	1.346~2.195	< 0.001			
*Gender*						
Male	1			1		
Female	0.832	0.671~1.032	0.094	0.657	0.437~0.989	0.044
*Race*						
White	1					
Black	0.967	0.632~1.479	0.876			
Other	0.958	0.571~1.609	0.872			
***Decade of diagnosis***	0.985	0.975~0.994	< 0.001			
***Grade***	1.447	1.183~1.769	< 0.001	1.736	1.207~2.495	0.003
*Histological type*						
Osteosarcoma, NOS	1					
Chondroblastic osteosarcoma	0.888	0.673~1.171	0.400			
Other	0.808	0.584~1.116	0.196			
*Tumor Stage*						
localize	1			1		
regional	1.588	1.167~2.161	0.003	3.329	1.312~8.449	0.011
metastasis	3.386	2.469~4.463	< 0.001	7.983	3.121~20.416	< 0.001
***Tumor size***	1.325	1.063~1.651	0.012			
*Surgery*						
Yes	1					
No	2.285	1.738~3.005	< 0.001			
*Chemotherapy*						
Yes	1					
No/unknow	0.932	0.733~1.186	0.568			
*Radiotherapy*						
Yes	1			1		
No/unknow	0.542	0.434~0.677	< 0.001	0.606	0.385~0.956	0.031

**Figure 1:**
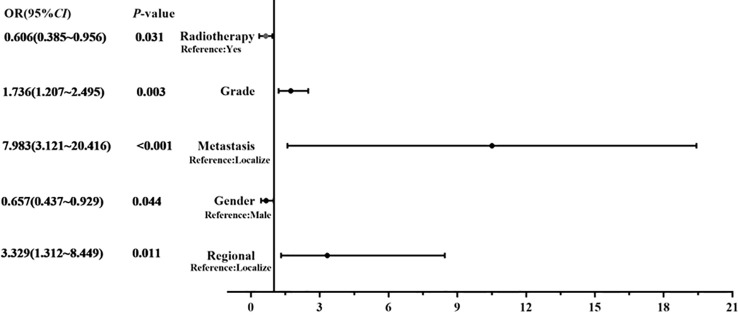
Forest map showing the independent risk factors of DDS in spinal osteosarcoma patients.

**Figure 2:**
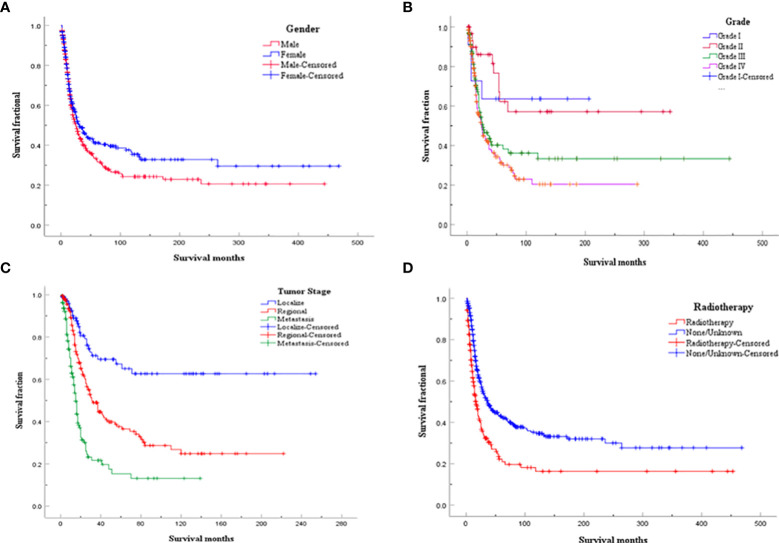
Kaplan-Meier analysis for DDS of spinal osteosarcoma according to **(A)** gender; **(B)** Tumor grade; **(C)** Tumor stage; **(D)** Radiotherapy.

Considering the limitations of univariate Cox analysis, multivariable Cox analysis was performed to investigate the independent prognostic factors associated with OS and DSS (**Tables 2**, **3**). As indicated by multivariate analysis of all spinal osteosarcoma patients, there was a negative correlation between OS and age ≥ 60 (HR = 2.271, *p* = 0.008), high grade (HR = 1.323, *p* = 0.008), regional stage (HR = 1.658, *p* = 0.017), metastasis stage (HR = 3.045, *p* < 0.001), and no-surgery treatment (HR = 1.761, *p* < 0.001). In terms of DSS, gender (HR = 0.657, *p* = 0.044), tumor grade (HR = 1.736, *p* = 0.003), tumor stage (HR = 3.329, *p* = 0.011; HR = 7.983, *p* < 0.001), and radiotherapy (HR = 0.606, *p* = 0.031) all were independent prognostic factors (**Figures 1**, **2**).

## 4 Discussion

Spinal osteosarcoma is a rare malignant tumor with susceptibility of invasive destruction and systemic metastasis, accounting for 3-5% of all cases of osteosarcoma and about 10% of primary spine tumors (15, 16). Although extensive resection and neoadjuvant chemotherapy have greatly improved the prognosis of patients with limb osteosarcoma (17, 18), there are still few large population-based studies to estimate survival and determine prognostic factors of patients with spinal osteosarcoma due to rarity of the disease. Therefore, identification of risk and prognostic factors of spinal osteosarcoma is of great clinical significance in early diagnosis, treatment and prognostic prediction of the disease.

To further understand spinal osteosarcoma, we conducted a comprehensive analysis by using the SEER database, aiming to estimate the survival of patients with spinal osteosarcoma, identify prognostic factors, and propose a standard treatment strategy. To the best of our knowledge, the current research is the largest retrospective research on spinal osteosarcoma involving a total of 668 cases diagnosed between 1975 and 2016.

It was found in this work that the mean age of these patients with spinal osteosarcoma was 45.2 years at diagnosis. There was no significant gender preference in patients with spinal osteosarcoma (M/F = 1.13:1), although male predilection was reported in some case reports in the previous literature (19). Regarding survival outcomes, the median OS of the patients with spinal osteosarcoma was about 15 months and the 5-year OS was about 16.8%, which are both worse than those of patients with limb osteosarcoma. This may be caused by the following reasons. First, spinal osteosarcoma is more susceptible to metastasis at diagnosis than limb osteosarcoma, probably due to the delayed diagnosis or the age-specific differences in tumor biology (20). Second, it is usually difficult to detect spinal osteosarcoma in the early stage because local pain is the first or even the only symptom, which is often mistaken for the symptom caused by some benign diseases, resulting in delayed diagnosis and high risk of metastasis (21). Third, although en-block resection is the most effective surgical method for the treatment of spinal tumors, radical surgery is more difficult for spinal osteosarcoma because of the complex anatomical structures and associated complications.

It is worth noting that patients above 60 years had median OS of 8 months and 5-year OS of 4.6%. Ours multivariate analysis showed that age was not a prognostic factor in DSS, but OS was decreased significantly in patients older than 60 years, which is consistent with the previous reports. This trend is understandable, given the aggressive treatment for such diseases. Like other literature reports, we also identified the male gender as an independent risk factor for worse DSS in patients with spinal osteosarcoma, which may be attributed to the aggressiveness of the tumor and/or poor response to treatment (13). Although advances in medical technologies have to some extent improved the overall clinical outcome of spinal osteosarcoma, our multivariate analysis showed no significant improvement in DSS over the past decade. Besides, our research identified osteosarcoma NOS as the most common histological type in spinal osteosarcoma, which is similar to previous studies on all osteosarcomas (13). Tumor grade and stage are generally recognized as the independent prognostic indicators of both OS and DSS in patients with spinal osteosarcoma. A high grade (low differentiation or differentiation) is known as the risk factor of mortality and is associated with higher rates of metastasis and recurrence as compared with the low grade (high or moderate differentiation) (22). Many other studies have reported that tumor size above 10 cm is associated with poorer prognosis and decreased survival in osteosarcoma patients (23–25). However, our study showed that tumor size had no significant value in predicting OS and DSS of patients with spinal osteosarcoma.

The multivariate analysis indicated that definitive surgery had a positive effect on OS in patients with spinal osteosarcoma, while chemotherapy did not seem to significantly affect either OS or DSS. Other studies also found that chemotherapy could not prolong the survival of osteosarcoma patients (26). Interestingly, our study discovered that radiotherapy tended to decrease OS and DSS, while there was no statistical difference as for OS in the multivariate analysis, which is not consistent with the previous research (27). Surgical resection and neoadjuvant chemotherapy are considered the standard treatments for osteosarcoma, but the efficacy of radiotherapy is still controversial. Radiotherapy does not seem to bring about significant benefits to patients with spinal osteosarcomas due to radiotherapy resistance and the risk of radiation-induced spinal cord injury and paralysis in most cases (28). Therefore, radiotherapy may be more commonly used for local control, or as a last resort in inoperable cases (29–31). In addition, as indicated by many studies (32–34), radiation can induce aggressive behavior in osteosarcoma, and therefore radiotherapy should be used prudently in these patients.

The limitations of this work need to be considered, and this is inherent to registration-based databases. Although the SEER is an excellent resource for the longitudinal and population data analysis and the surgical intervention condition report, there are still limitations in its ability to retrospectively analyze other surgical variables, such as surgery type, margin status, surgical resection extent, and postoperative tumor recurrence. Besides, the SEER database does not contain sufficient information on specific chemotherapy regimens, radiation therapy dose, and molecular pathological characteristics, which may affect the prognosis of patients, and these variables may be an effective complement to this work. Furthermore, it is inevitable that data of some patients may be lost, due to the retrospective characteristic of this work. This may reduce the number of eligible cases. Despite these limitations, to our knowledge, the current research represents the largest analysis evaluating the important associations and predictors of spinal osteosarcoma.

## 5 Conclusion

In this population-based research, we made a comprehensive analysis of the clinical characteristics of spinal osteosarcoma, aiming to improve the current awareness and diagnosis of this malignant tumor. The male gender, a high tumor grade, regional stage and metastasis stage, and radiotherapy are independent predictors of poor survival of patients with spinal osteosarcoma. Treatment of spinal osteosarcoma remains a clinical challenge at present. In addition to the conventional treatments, future research should devote more efforts to gaining a better understanding about the pathogenesis of spinal osteosarcoma and explore the clinical impact and survival outcomes of the emerging targeted and immune therapies for this rare and aggressive disease.

## Data availability statement

The datasets presented in this study can be found in online repositories. The names of the repository/repositories and accession number(s) can be found in the article/supplementary material.

## Ethics statement

Ethical review and approval was not required for the study on human participants in accordance with the local legislation and institutional requirements. Written informed consent for participation was not required for this study in accordance with the national legislation and the institutional requirements.

## Author contributions

T-lL: conceptualization, supervision, and methodology. JW: formal analysis, investigation, and writing-original draft preparation. X-zN: data curation and writing- reviewing and editing. M-lY: data curation and investigation. S-mH: investigation. XH: investigation. CP: investigation. J-sC: investigation. All authors contributed to the article and approved the submitted version.
